# Individually customisable non-invasive head immobilisation system for non-human primates with an option for voluntary engagement

**DOI:** 10.1016/j.jneumeth.2016.05.009

**Published:** 2016-08-30

**Authors:** Heather Slater, Alice E. Milne, Benjamin Wilson, Ross S. Muers, Fabien Balezeau, David Hunter, Alexander Thiele, Timothy D. Griffiths, Christopher I. Petkov

**Affiliations:** a Institute of Neuroscience, Henry Wellcome Building, Newcastle University, Framlington Place, Newcastle upon Tyne NE2 4HH, United Kingdom; b Centre for Behaviour and Evolution, Henry Wellcome Building, Newcastle University, Framlington Place, Newcastle upon Tyne NE2 4HH, United Kingdom

**Keywords:** Head immobilisation, Non-invasive, Macaque, Monkey, Eye tracking, MRI, Animal welfare

## Abstract

**Background:**

Head immobilisation is often necessary for neuroscientific procedures. A number of Non-invasive Head Immobilisation Systems (NHIS) for monkeys are available, but the need remains for a feasible integrated system combining a broad range of essential features.

**New method:**

We developed an individualised macaque NHIS addressing several animal welfare and scientific needs. The system comprises a customised-to-fit facemask that can be used separately or combined with a back piece to form a full-head helmet. The system permits presentation of visual and auditory stimuli during immobilisation and provides mouth access for reward.

**Results:**

The facemask was incorporated into an automated voluntary training system, allowing the animals to engage with it for increasing periods leading to full head immobilisation. We evaluated the system during performance on several auditory or visual behavioural tasks with testing sessions lasting 1.5–2 h, used thermal imaging to monitor for and prevent pressure points, and measured head movement using MRI.

**Comparison with existing methods:**

A comprehensive evaluation of the system is provided in relation to several scientific and animal welfare requirements. Behavioural results were often comparable to those obtained with surgical implants. Cost–benefit analyses were conducted comparing the system with surgical options, highlighting the benefits of implementing the non-invasive option.

**Conclusions:**

The system has a number of potential applications and could be an important tool in neuroscientific research, when direct access to the brain for neuronal recordings is not required, offering the opportunity to conduct non-invasive experiments while improving animal welfare and reducing reliance on surgically implanted head posts.

## Highlights

• Non-invasive head immobilisation for neuroscience experiments in monkeys.
• Individually customised system combining functionality of previous systems.
• Allows access for auditory and visual stimulation.
• Has the option for voluntary engagement to assist habituation.
• Systematically evaluated against scientific and animal welfare needs.

## 1 Introduction

The present report describes the development and systematic evaluation of a non-invasive alternative to the use of surgically implanted head posts for use with macaque monkeys, a laboratory animal commonly used as a neurobiological model to advance our understanding of human neurobiology and disorders of the nervous system. Many neuroscientific procedures involving animals require head immobilisation. Typical approaches use an implanted head post, which is attached to the skull of the animal during an aseptic surgical procedure under general anaesthesia (Betelak et al., 2001, Mountcastle et al., 1975). In addition to limiting head movement, the implant can accommodate chambers used for direct neuronal recordings. However, surgical implants carry a risk of infection and can become unstable or fail. If this occurs and the animal cannot be re-implanted, further data collection may not be possible and the animal would need to be replaced. Thus, for approaches that depend on minimal head movement but do not require direct access to the brain, Non-invasive Head Immobilisation Systems (NHIS) could prove beneficial in reducing the reliance on surgical implants. However, if NHIS are to be broadly accepted as viable alternatives they need to address combinations of scientific and animal welfare requirements and show comparable data quality in relation to surgical implant approaches.

We aimed to contribute to the ongoing effort to develop and refine non-invasive head immobilisation options, identifying several scientific and animal welfare considerations. We summarise recent NHIS against eight criteria shown in Table 1. This shows that most recent systems are individually customisable to better fit the animal’s head, however, surprisingly little is known about how the systems impact on levels of distress or discomfort experienced by the animals during habituation to or use of the system. Facemasks have been used to allow an animal to voluntarily engage with an experimental setup for eye tracking and measurement (Fairhall et al., 2006, Kiorpes et al., 2012), see ♦ in Table 1, rows 8–9. It may be useful to implement an automated system to allow the animals to voluntarily engage with the facemask at their leisure, which could help them to habituate to full head immobilisation (Table 1, rows 1–7), but this is currently not available as an option. Moreover, it remains unclear the extent to which pressure points form during immobilisation, or how this is monitored and addressed if pressure points do occur, in order to alleviate pain or prevent sore formation and infection. Also some systems block access to the ears for high-fidelity auditory stimulation and it remains unclear how adaptable the systems are to different types of laboratory settings, since most are often demonstrated within a single setup. Lastly, it is important that any system is robust and shown to work with animals of different sizes.

To address these needs, we designed and evaluated a system that combines the essential features of the available systems while also extending the range of features. This effort resulted in a system that has considerable flexibility in how it is used, which, to our knowledge, for the first time incorporates an option for automated voluntary engagement with the facemask as an initial step towards the animals habituating to immobilisation using the full-head helmet. We comprehensively evaluated the system against the specified criteria within the context of documented behavioural habituation and training steps as several animals were trained to use the system. We further assessed performance on challenging auditory tasks. Some of the results are also directly compared with those from the animal’s own surgically implanted head posts. We also provide a cost–benefit analysis to help others assess the desirability of such a system for applications in other laboratories. The findings, in many cases, bode well for this system as a practical comprehensive approach for non-invasive head immobilisation that is not overly time consuming to implement and as a relatively low cost alternative to surgically implanted options where direct access to the brain is not required.

## 2 Methods

### 2.1 Subjects

All of the animal procedures performed were approved by the UK Home Office and comply with the Animal Scientific Procedures Act (1986) on the care and use of animals in research and with the European Directive on the protection of animals used in research (2010/63/EU). We support the Animal Research Reporting of In Vivo Experiments (ARRIVE) principles on reporting animal research. All persons involved in this project were Home Office certified and the work was strictly regulated by the U.K. Home Office.

Seven male rhesus macaques (*Macaca mulatta*) from a group of pair housed animals were used for the development and evaluation of the system described here. The pen sizes in our colony range from 130 × 240 cm to 215 × 240 cm. All are 230 cm high, and hatches between neighbouring cages are used to increase the space available to the animals. One monkey (M1, 5 years, 12 kg) was naïve to behavioural and head immobilisation training, not having previously had an implanted head post. Two other monkeys (M2, 11 kg; M3, 16 kg, both 8 years old at the time of testing) did not have implanted head posts at the time of assessment, but had previously had head post implants. These head posts had become unstable and were removed at 7 months and 4 years after implantation, respectively. The other animals (M4, 6 years, 12.5 kg; M5, 6 years, 14 kg; M6, 8 years, 15 kg; M7, 4 years, 6 kg) had existing implants, allowing direct comparison between implanted animals and those using the NHIS. Table 2 summarises the procedures conducted using the head immobilisation device with each animal in this report.

### 2.2 General design features of the nonhuman primate, non-invasive head immobilisation system

In collaboration with the Freeman Hospital Cancer Radiotherapy Unit at Newcastle upon Tyne, UK, we prototyped and developed a non-invasive head immobilisation system for nonhuman primates, using similar design approaches as those in use in human radiotherapy cancer treatment units. In developing the nonhuman primate NHIS, we combined the experience of the Freeman Hospital Unit in developing and using highly customised whole head or limb immobilisation in human patients with our experience working with nonhuman primates on neuroscientific procedures.

**Fig. 1:**
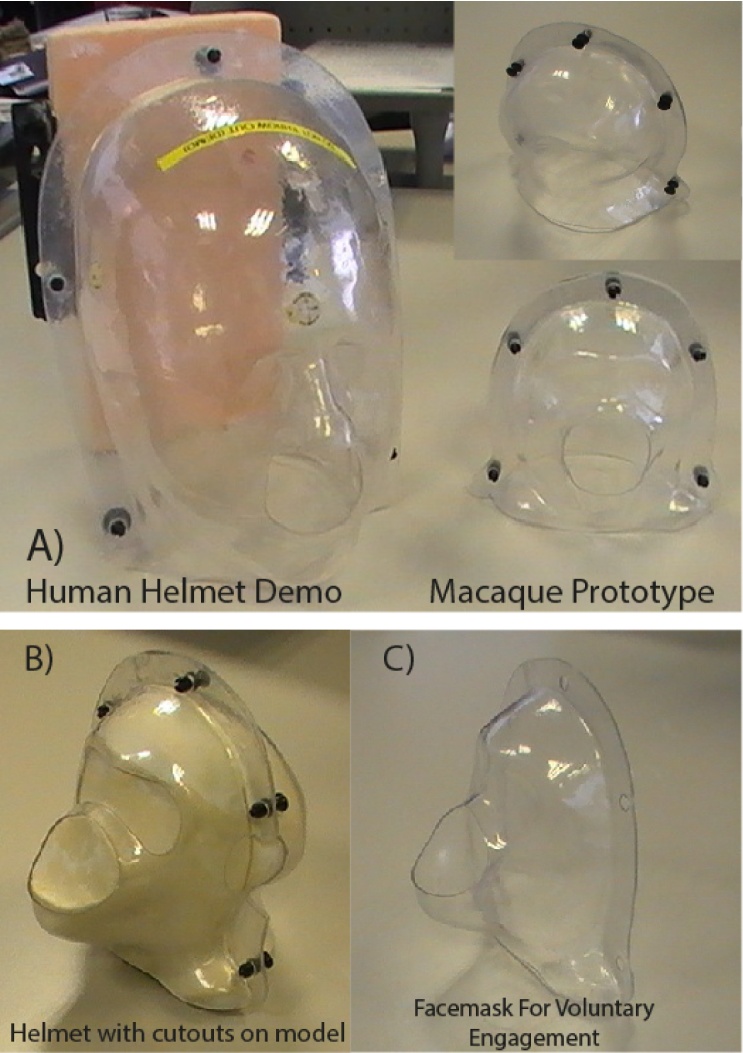
A monkey non-invasive head immobilisation system, based on head immobilisation methods used with human head or neck cancer patients being treated with radiotherapy. (A) Shows a transparent demo of a human helmet that is customised for a human cancer radiotherapy patient. To the right is shown the prototype that was developed here for neuroscientific research with macaques. (B) The transparent plastic helmet can be easily modified to include cut outs allowing the animal to see, hear and make small mouth movements to drink fluid rewards. (C) The two piece helmet system can be separated so that the facemask can be used alone for initial habituation training to whole head immobilisation, with periods of voluntary immobilisation of the animal.

The system was designed to achieve head immobilisation for macaques of different sizes providing a highly customised fit and allowing for visual and auditory stimulation and for the animals to receive fluid rewards as positive reinforcement (Fig. 1). The transparent plastic allows the animals to see through the facemask while it is being placed, which makes placement of the facemask less intimidating or distressing. The plastic can be greatly modified while retaining structural strength; air holes can be created and the plastic can be thinned in problem areas to prevent overheating and to alleviate pressure points. It can be easily modified to incorporate fittings for a wide range of scientific and laboratory attachments, which can readily be integrated into the facemask or full-head helmet system.

### 2.3 Creating the head model

We used two different methods to create a head model from which the helmet system could be made.

#### 2.3.1 Head impression using plaster bandages and alginate

For one approach, we created an impression of the whole head using plaster bandages and alginate moulding putty (BabyRice Chromatic Alginate Moulding Material mixed with water). The head impression was filled with plaster to create the head model. First the animal was sedated, e.g., with ketamine (0.1 ml/kg; Henry Schein, trade name Narketan 10) while blood oxygen saturation was monitored and maintained by providing additional oxygen if needed, and ensuring that the breathing pathways were unobstructed. The eyes were protected by closing the eyelids and covering them with gauze and plastic cling film. Excess hair was trimmed from the areas to be moulded and aqueous cream was applied to prevent the impression material from sticking to remaining hair. The mould of the back of the head was made by gently lowering the animal’s head into a suitable container, containing a cutaway section to accommodate the neck, filled with alginate to take the impression. Plaster bandages were applied to the face and allowed to set, ensuring the mouth and nose were not obstructed.

This procedure took about 15–25 min to complete. Once the bandages and alginate had set, they were removed and any remaining cream or moulding material was removed by hand from the monkey’s head and neck. The animal was then monitored in a recovery unit until fully conscious before it was returned to the home cage.

**Fig. 2:**
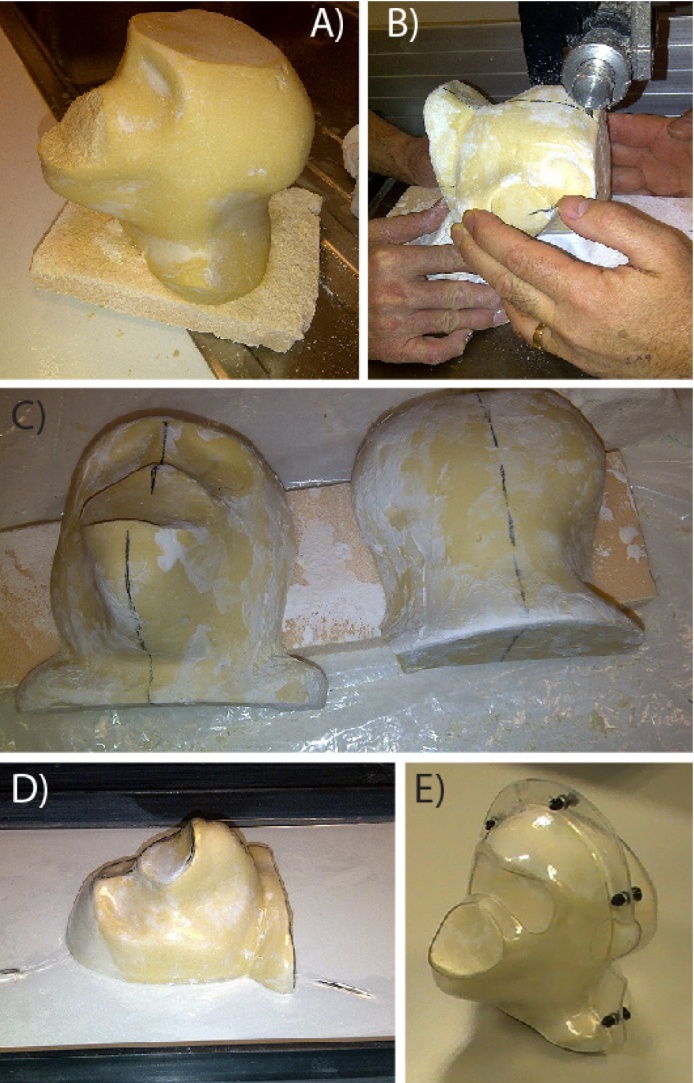
Creating the thermo-plastic shell for the helmet. A model of the animal’s head is first created, see text. (A) The model is then halved to create a face and back piece as shown (B and C). Thermal plastic is heated and moulded around the head model using a vacuum forming machine (D). A hand drill is used to cut out areas for the ears, eyes and mouth and to place attachments as needed (E).

The plaster facemask and alginate back piece were joined together using additional bandages. Plaster of Paris was then poured into the impressions and allowed to set to create the head model. Following setting of the plaster, the bandages were removed and the rough edges of the head model were filed down. The helmet was then created by halving the model (as shown in Fig. 2) and placing each half into a vacuum forming machine (C.R. Clarke Vacuum Former 1210). Sheets of 4 mm thick Polyethylene terephthalate (PETG) thermoplastic (Bay Plastics) were heated in the machine and these were vacuum formed around the head model. A hand held rotary tool (Dremel) was used to trim excess plastic from the shell and shape the front and back pieces of the helmet as desired. The two halves can additionally be made in isolation rather than producing a full model. For example, once a full helmet has been created, if only a new front or back piece is needed, the other half can be used to position the animal while an impression is retaken of the desired area. However, for greatest accuracy in creating the initial model we recommend creating the whole head impression. Straws inserted into the alginate impression either side of the head can help in realigning the front and back pieces after the alginate sets and the impression is taken.

Additionally, we have used alginate putty to improve the fit of helmet pieces. For example, if the head immobilisation requires adjustment to further refine the fit, alginate putty can be applied to the inside of an existing mask. The mask plus the alginate putty on the inside of it are then placed over the animal’s head under sedation. Once the alginate has set, the facemask is removed and the set alginate is left inside the plastic. This can then be used to create a new model of the head to create a better fitting or more accurate facemask for the helmet. A similar procedure can be done to improve the fit of the back piece.

#### 2.3.2 Head model creation using MRI

We have also acquired a model of the head using MRI imaging under anaesthesia. For each procedure the animal was initially sedated with ketamine (i.m. 10 mg/kg) before being pre-oxygenated and prepared for intubation (Propofol, typically 3–4 mg/kg i.v.). The trachea was intubated and the lungs ventilated (at 25 strokes/min) to maintain expired CO_2_ within the physiological range. Anaesthesia was maintained with sevoflurane 2.5–3.0% mixed with 100% oxygen. Lactated Ringer’s solution was given intravenously at a maximum rate of 10 ml/kg/h. Physiological parameters (heart rate, blood pressure, blood oxygenation, and expiratory CO_2_) were monitored and kept in desired ranges with volume supplements. When the animals were fully anaesthetised, they were then transferred to a primate MRI scanning chair, where they were held in place using body supports, ear bars and padding to support the head.

**Fig. 3:**
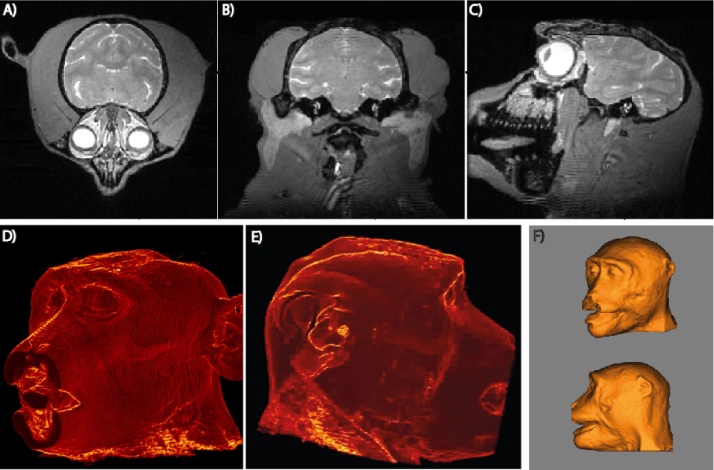
Acquiring a head model using MRI. A whole head MRI is taken of the animal (A–C) and converted into a 3D surface using Amide 3D software (D–F; shown for M1, M2). The head 3D image is separated into two halves in post processing and the image files were sent to Rogue Research Inc. for 3D printing or printed locally on a 3D printer to create the model of the head. Images D and E taken from Supplementary Video 1.

MRI-based T1 weighted Modified Driven Equilibrium Fourier Transform (MDEFT) and T2 weighted Rapid Acquisition with Relaxation Enhancement (RARE) structural images of the whole head were taken on a nonhuman primate dedicated, vertical 4.7 T research MRI scanner (Bruker BioSpin, Ettlingen, Germany). The processed scans were converted into Analyze format to be loaded into a Medical Image Data Examiner (AMIDE, SourceForge, Slashdot Media; Fig. 3) and converted into 3D format. These were then 3D printed (Rogue Research Inc. or in-house) to produce the head model. As before, the model was halved and each half was used to create the thermoplastic shell of the helmet on a vacuum forming machine.

Creating a model using plaster bandages is relatively easy and cheap to perform and does not require general anaesthesia or complex imaging techniques. It could therefore be used in most primate labs. The second approach has the potential to provide more anatomically accurate head models, but is more demanding in terms of equipment and resources. A similar procedure as the one described for creating the head model using MRI, but instead using Computerised Tomography could in principle also be used if the equipment is available. Generally, we found that the models created with either the plaster bandages or MRI provided sufficiently realistic models of the animals’ heads for creating the NHIS.

### 2.4 Design of the facemask and helmet system for use in the laboratory

**Fig. 4:**
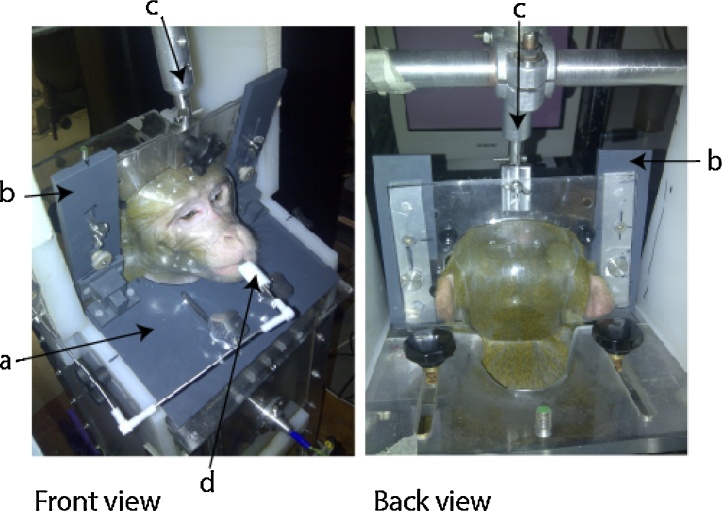
The helmet system within a typical laboratory working chair. The front of the transparent mask is fitted to the frame (a and b) and attached to the training chair. The initial attachment is through the base plate of the frame (a). The brackets to the side (b) are fixed via hinges which allow the mask to be tilted as needed. The head bar attachment (c) can provide further rigidity. The animal receives reward for his task through a juice reward system and can breathe, drink and move its lips (d). Several air holes can be added to the helmet to help with ventilation. The translucent facemask and back piece provide an individually customised fit for each animal.

**Fig. 5:**
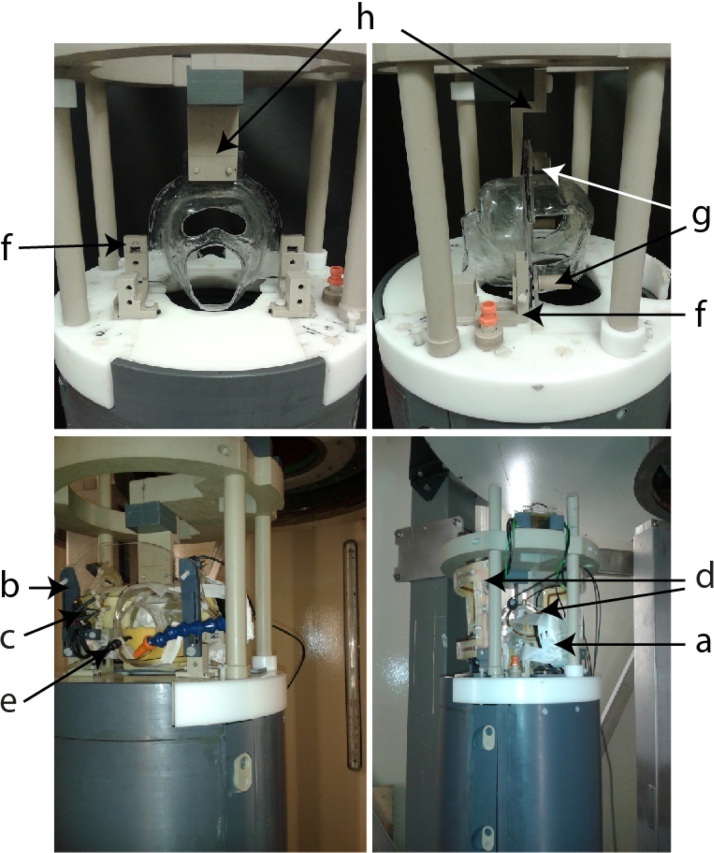
Alternative setup for MRI data collection in a vertical primate-dedicated MRI scanner. Removal of the plastic surrounding the face and head pieces allows for more room around the head. This accommodates placement of headphones (a), mirror (b), camera (c) and coils (d) needed for scanning, and the juice tube (e) needed for providing reward. The mask is initially secured to the chair with customised fittings (f) before the back piece is attached via thumb screws (g) and the helmet is secured to a point at the top which is where an implanted head post would usually be fixed (h). We have implemented this for use with our single and multi-channel (shown here) MRI imaging coil setups.

**Fig. 6:**
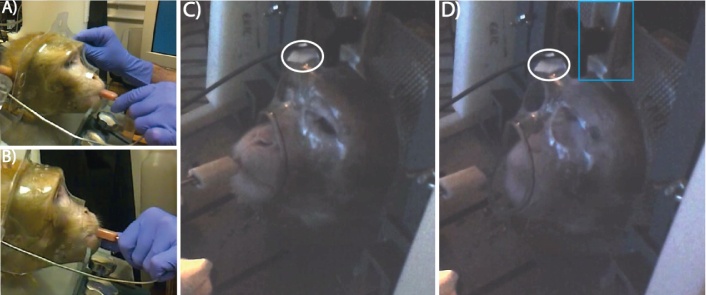
Voluntary facemask engagement during initial habituation training. Initially the animal is introduced to the mask and receives reward while placing his face inside the customised facemask (A and B, M2). The face mask is then attached to the training chair (C and D, M3) with extra stability for the helmet system provided by the head bar usually used for attaching to a surgical implant, as an option (indicated with a blue box in D). An infra-red sensor is placed at the top of the mask (indicated with a white circle) which is activated by the presence of the animal’s face in the facemask. On sensor activation, juice is dispensed via the reward system. Following this, the sensor is placed on the back piece of the helmet system and the animal is rewarded when it keeps its face in the facemask with the back piece touching its head, until the back piece is completely attached. Images shown here are frames from Supplementary Videos 2–3. (For interpretation of the references to colour in this figure legend, the reader is referred to the web version of this article.)

The helmet system comprises a mask and a back piece which is placed over the back of the head. Initially the animal is seated in a standard vertical primate training chair and restrained via the neck plate attached to the chair (see Fig. 4, Fig. 5, Fig. 6 ). No additional restraint is used. The facemask is attached to the training chair via a frame which fits over two attachment bolts protruding from the neck plate. Thumb screws are used to secure the facemask to the bolts in the chair (Fig. 4). The frame can be moved back and forth and has hinges which allow the mask to be tilted to accommodate the natural position of the monkey’s head. The facemask is then secured in the desired position. The back piece attaches from behind to meet with the front piece and is secured to the frame via thumb screws which fit through the back and front of the device. Plastic snaps can also secure the two pieces. Additional rigidity is achieved by incorporating the metal head bar attachment which is commonly used in laboratories to attach to the animal’s surgically implanted head post, but in this case it is attached to the helmet. Using the metal bar to provide additional stability to the system from above is not critical, but can provide additional stability when the facemask is used alone, e.g., for voluntary engagement (see Fig. 6). During training the animal receives reward for correct task performance through the mouth piece. The mouth cut out allows it to breathe, drink and move its lips.

In our initial training setup the remaining plastic sheet left after forming the facemask/helmet was retained as desired to use it to attach the facemask/helmet to the frame of the chair. The surrounding plastic, can be removed or flexibly modified to allow attachment of the system as needed for different laboratory setups. We have additionally implemented the system on our primate dedicated vertical bore MRI scanner chairs, which is more specialised laboratory environment, using attachments made specifically from MRI compatible polyether ether ketone (PEEK) material. These attach to the top of the mask in a similar way as the metal head post holder in the laboratory setting where using ferromagnetic materials is not an issue. For MRI all ferromagnetic materials need to be replaced with non-magnetic materials such as PEEK plastic. The bottom of the mask is given extra support by two MRI compatible PEEK legs, which attach the base of the mask to the chair (Fig. 5). The system could in principle also be flexibly modified for use in a horizontal bore scanner.

### 2.5 Voluntary facemask engagement training for auditory behavioural and simple eye fixation tasks

We assessed performance on two different auditory behavioural tasks during voluntary engagement. The front piece of the helmet system (the facemask) was initially used to gradually habituate monkeys to head immobilisation.

**Fig. 7:**
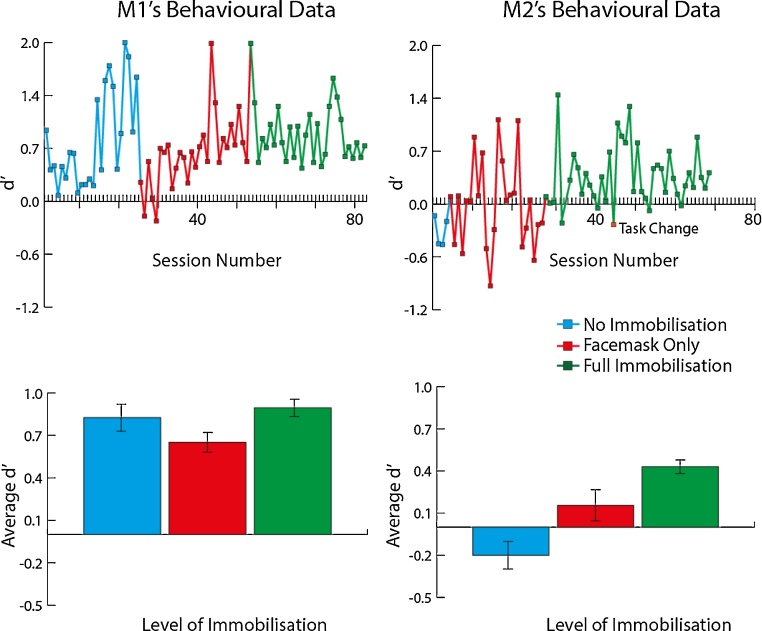
Performance on an auditory spatial discrimination task during different stages of habituation to facemask or helmet systems. Average *d*′ during the testing session was used to assess performance on the task for these two monkeys (M1, M2).

Two of the animals (M1 and M2) were trained to perform an auditory spatial discrimination task, initially without any head immobilisation. Sounds were presented from two speakers placed on either side of a computer monitor and the monkeys were trained to hold a touch lever inside the chair and release the lever whenever they detected that the second of two sounds presented in a sequence was in a different spatial location in relation to the first sound (spatial location change between ±90°). The animals were given a reward if they correctly detected the spatial location change indicated by a lever release (hit) or refrained from releasing the lever when the sound did not change in spatial location (correct rejection). No reward was delivered and a time delay was introduced when a false alarm (the animal released the lever when the sounds did not change in spatial location) or a miss occurred (the animal missed the change in spatial location and did not release the lever). Performance on the task was measured by calculating *d*′ (Green and Swets, 1966): *d*′ is a measure of sensitivity, in this case between moving and static auditory stimuli, calculated using the proportions of hits and false alarms. Increasing values correspond to greater levels of behavioural sensitivity to the target stimuli, with a *d*′ of zero reflecting a lack of sensitivity. Negative values are also possible when an animal produces more false alarms than hits (as might happen during initial stages of training, see the variability in the early performance of M2 in Fig. 7).

Early in training, the facemask alone was attached to the front of the chair and voluntary engagement training began. The animals performed their task while engaging with the mask in order to receive reward for correct trial completion. Over the course of 4–5 testing sessions the mask was moved closer to the animal’s face to encourage voluntary engagement. Following this, the animals performed their task with the facemask fixed in place. Initial training on the task did not require complete head immobilisation, but as the animals were required to identify a change in direction of a sound, it was important to have them face forwards and engage with the facemask for more accurate perception of the spatial location change in the stimuli. Full head immobilisation was later required for the placement of headphones for more accurate stimulus presentation and for habituation to other aspects of the scanner setup. Once performance was stable and the animal was willing to place their face into the mask, the back piece was gradually introduced and fixed in place. Initially the back piece was held by hand over the back of the animal’s head. Once this was tolerated, the back piece was attached to the front piece loosely so that some movement was possible, but not enough for the macaque to fully remove their face from the mask. Finally the back was attached for increasing lengths of time with full head immobilisation.

A third animal (M3) had been trained to perform basic eye fixation prior to the loss of his implant at age 8 years (for methods on eye fixation training used for this animal, see Wilson et al., 2013). To assess the relative quality of eye-tracking data using this NHIS, we gradually habituated the monkey to the facemask using voluntary engagement. We proceeded with habituation training using an infra-red proximity sensor (OPB733TR; OPTEK Technology) which was placed on the outside of the mask. When the sensor was activated by the presence of the animal’s face in the facemask, juice reward was dispensed. The reward was then delayed, e.g., 1000–2000 ms, to encourage them to hold their face within the mask for longer periods. The reward can also be delivered continuously for as long as the monkey’s face is present in the mask and stopped when the sensor detects that they have removed their face (see Supplementary Video 3). Once the animal readily engaged the mask, we relocated the sensor to the back piece of the helmet and repeated this procedure while placing the back piece over the back of the head as the animal engaged the facemask. We then reduced the level of free movement slowly during full helmet attachment. Following this, eye fixation training was able to resume.

For comparative data collection, we used the same experimental design as had been used for a previous experiment involving M3 performing a fixation task (Wilson et al., 2013), using an infra-red eye tracking system (Arrington Research). Briefly, M3 was seated in a primate chair 60 cm in front of a computer monitor. A fixation spot was displayed at the centre of the computer monitor and he was rewarded for visually fixating on it for 4 s within a fixation window of 5° visual angle. Trials in which he failed to fixate on the spot for 4 s were classed as aborts and were restarted after a brief inter-trial interval. In the initial head-posted experiment auditory stimuli were infrequently (25% of trials) presented from audio speakers located to the left or right of the computer monitor. We used the same experimental setup for data collection with the NHIS, however, no auditory stimuli were used. For the analysis, only trials for which no additional stimuli were presented in the original experiment were used; therefore the data represent a 4 s fixation period and a 3 s period during which no auditory or visual stimuli were presented and the animal was free to look around. In the second experiment using the helmet, the monkey was again presented with a fixation spot for 4 s following which eye-tracking data was recorded for an additional 3 s in the absence of any other stimuli. In both experiments 10 testing runs, each containing 16 trials, were collected.

## 3 Results

### 3.1 Task performance and behaviour during voluntary engagement and non-invasive head immobilisation

Performance on an auditory spatial discrimination task (see Section 2) was measured over the different stages of habituation to the system, as shown in Fig. 7. The initial stage of training with M1 and M2 involved no immobilisation. The facemask alone was then introduced for voluntary engagement training in combination with task performance, and this was followed later by attaching the back piece of the helmet. Fig. 7 shows that performance on the task improved or remained stable throughout the three stages. In M1, who was already performing at a good level, performance was relatively stable across the different procedures (no significant difference in behavioural performance across the conditions; ANOVA; *F*_2,66_ = 2.19, *p* = 0.12; Fig. 7). M2’s performance significantly improved during the facemask and helmet immobilisation procedure (*F*_2,79_ = 8.66, *p* < 0.001), as the animal learned and got better at the task. Thus, there was no overall detrimental effect on auditory task performance by implementation of the non-invasive head immobilisation procedures, demonstrating that this is an effective method for training macaques and collecting data on auditory spatial location tasks.

**Fig. 8:**
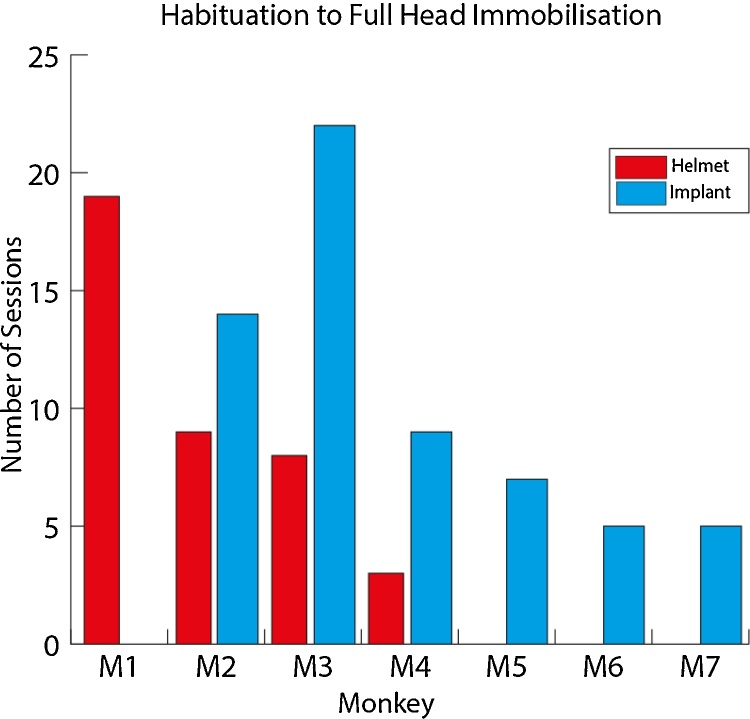
Initial habituation period for the two immobilisation methods: helmet versus implant. Monkeys M2, M3 and M4 had previously been trained with head immobilisation using their implanted head post. (For interpretation of the references to colour mentioned in the text, the reader is referred to the web version of this article.)

We also summarise the period of habituation to achieve full head immobilisation as the number of daily testing sessions required from the point at which we began to immobilise the animal’s head in any way, to the point at which they work for a full training session head immobilised (>30 min). For the animals with a surgical implant this refers to touching or holding the head post to allow the animal to become accustomed to movement restriction. For the animals using the helmet system, this refers to the point of initial introduction of the back of the helmet. For the helmet system (red bars in Fig. 8) full immobilisation was achieved in 3–19 daily testing sessions (mean = 10; standard error mean, SEM = 3.4) and for the implant in 5–22 sessions (mean = 10; SEM = 2.7). Fig. 8 suggests that habituation to the non-invasive system with full head immobilisation without distress requires at least as much time as habituating the animal to head immobilisation using an implanted head post, not including habituation training with the facemask which can take an additional ∼5 sessions.

### 3.2 Thermal imaging to monitor for hot-spots

**Fig. 9:**
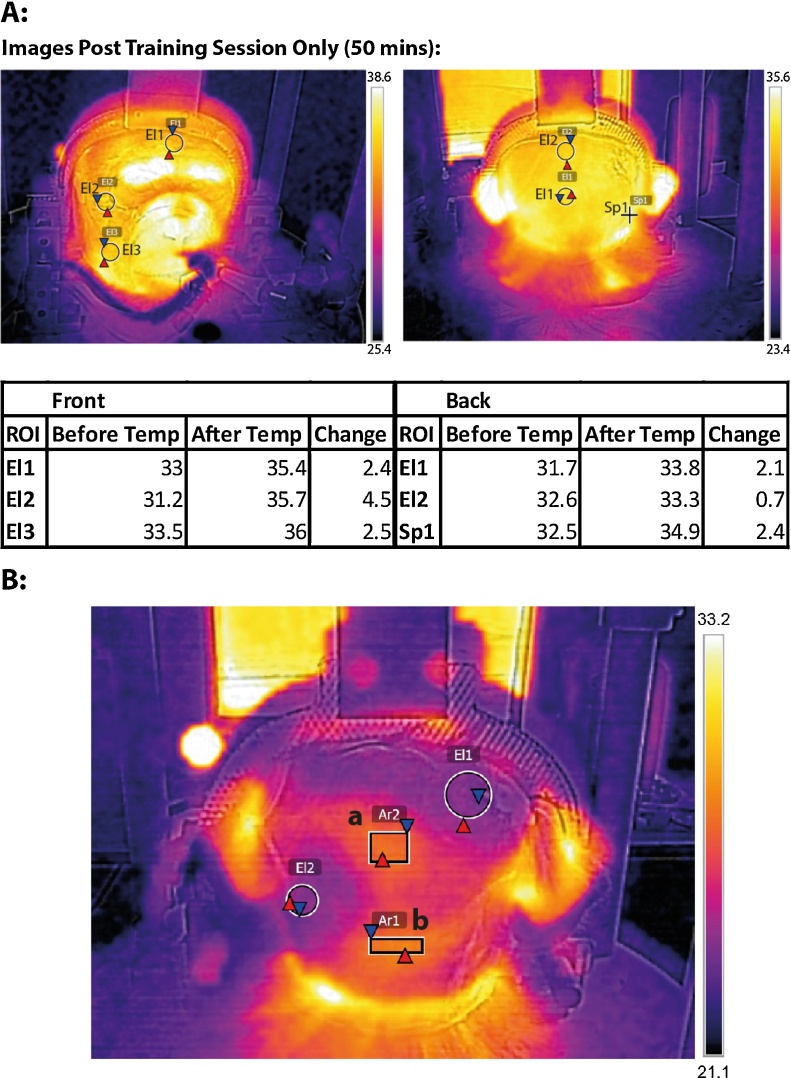
Thermal imaging to identify hot spot formation. (A) Thermal measurements before and after training. Images were taken of the face and back of the head before and after training (50 min of immobilisation at room temperature). For brevity, only post training images are shown. Hotspots can be identified and labelled (B: rectangles a and b) and ventilation holes placed in the plastic at those points to allow for better ventilation of the area and less pressure on the underlying part of the head/face. All temperatures reported are in degrees (°) Centigrade.

An area of increased pressure or contact between the animal’s head and the plastic can result in an increase in temperature or a “hot spot” in that area, which if not properly ventilated or depressurised could become sore and potentially infected. Being able to measure hot-spot formation in head immobilisation systems could identify potential problem areas that can be addressed by thinning or removing the plastic in that area, provided that the remaining pressure between the head and the immobilisation device is well distributed over a relatively large remaining area (Fig. 9B). We used an infra-red thermal imaging system (FLIR Systems E4 camera, FOL7 lens with 80 × 60 IR resolution) to assess the potential for the formation of hot spots. Readings were taken before and after a training session and the images were processed with the FLIR software to identify areas of increased temperature. These images were then used to guide the placement of ventilation holes in the plastic, if needed. We continued to monitor the effectiveness of these modifications in dissipating heat over the course of subsequent training sessions (Fig. 9A). Fig. 9 shows that heat within the helmet can increase within a range of 0.7–4.5 °C from the beginning to the end of the testing session (in this case 50 min). We suggest monitoring any spots that increase more than 3 °C during the course of the session and modifying the helmet system in those areas to decrease potential discomfort and reduce pressure point formation. A full helmet system like ours can accommodate many ventilation air holes without reducing stability in head immobilisation or rigidity of the system.

### 3.3 Numbers of sedations

**Fig. 10:**
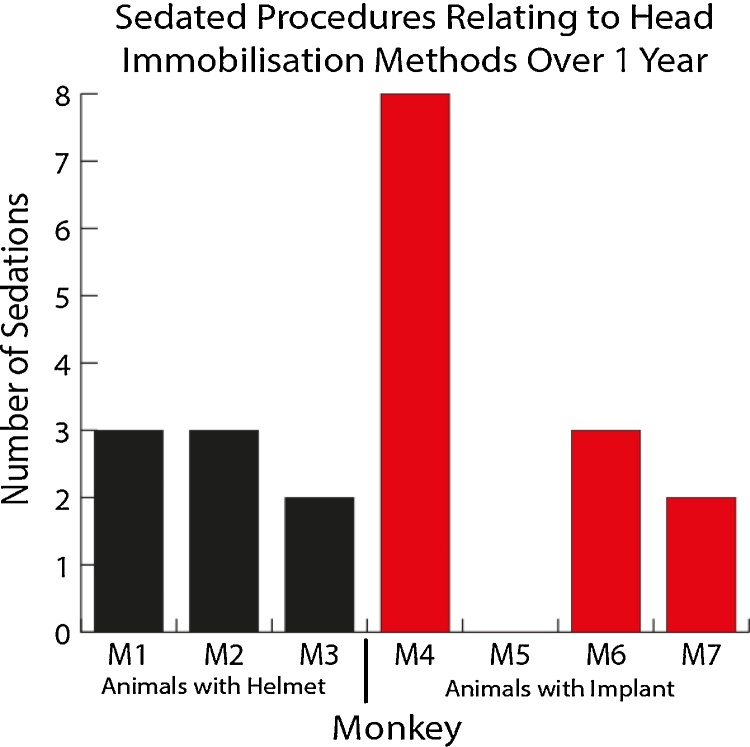
Number of sedations for each of the seven animals in this study during a one year period for immobilisation related procedures with the helmet system or to maintain surgical implants. For animals with an implant this refers to implant procedure (if it occurred within the year of monitoring) and sedations required for margin debridement (M4–M6 had their implant procedure in a previous year). For animals without a surgical implant this refers to sedations for obtaining head impressions or MRI based models of the head. Some animals, like M4 were sedated more regularly for implant debridement and maintenance procedures if these would distress the animal to conduct while it was awake.

Short term sedation with ketamine (0.1 mg/kg, ca. 30 min) is required both for the maintenance of a surgical implant and for obtaining a model of the head to produce the non-invasive head immobilisation system. For the MRI procedure general anaesthesia is required (initial sedation with ketamine followed by propofol and sevoflurane, ca. 1–2 h; see Section 2). If the animal grows, loses weight or the helmet becomes uncomfortable, the helmet may need to be replaced, requiring another procedure under sedation to obtain a new head model. We measured the numbers of sedation procedures across animals with surgical implants or for use with the non-invasive system. The number of occasions when an animal was sedated for implant maintenance or for head model creation is shown in Fig. 10. The figure shows that over the course of a year with these 7 macaques 2–3 sedations are needed for the non-invasive system, and anywhere between 0–8 sedations for surgical implant maintenance.

### 3.4 Number of helmet replacements over a 1 year period

**Fig. 11:**
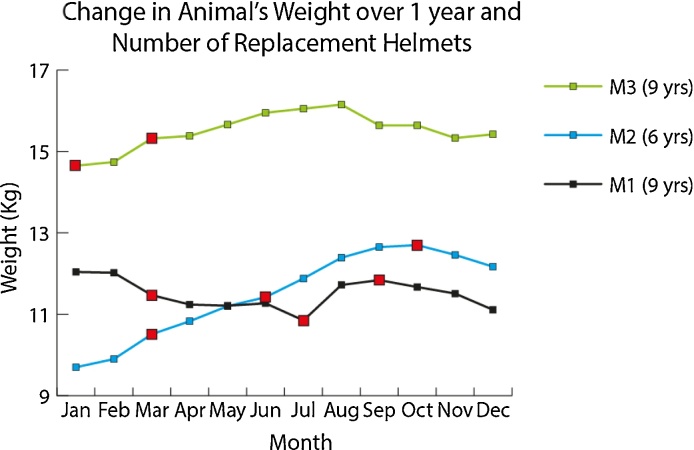
Weight change of animals and number of occasions when each animal had a helmet replacement during a one year period. Red markers indicate the point at which the helmet was replaced. As the weight of the animal changes the helmet/facemask may need to be adapted to eliminate discomfort, in the case of weight gain, or to improve the fit, in the case of weight loss. (For interpretation of the references to colour in this figure legend, the reader is referred to the web version of this article.)

An increase or decrease in weight of the animal has the potential to impact on the fit of the helmet. If the animal begins to display signs of discomfort such as reluctance to engage with the helmet, or the level of immobilisation provided is insufficient, it may be necessary to update the model of the head and produce a new facemask and helmet. Over the course of a year, three animals on study with the helmet system required 1–3 replacements, even in animals whose weight was relatively steady (Fig. 11). This highlights that the system might need updating ∼2 times a year and that body weight can, but does not always, predict when an animal might be due for a helmet replacement.

### 3.5 Simple eye fixation stability during head immobilisation

**Fig. 12:**
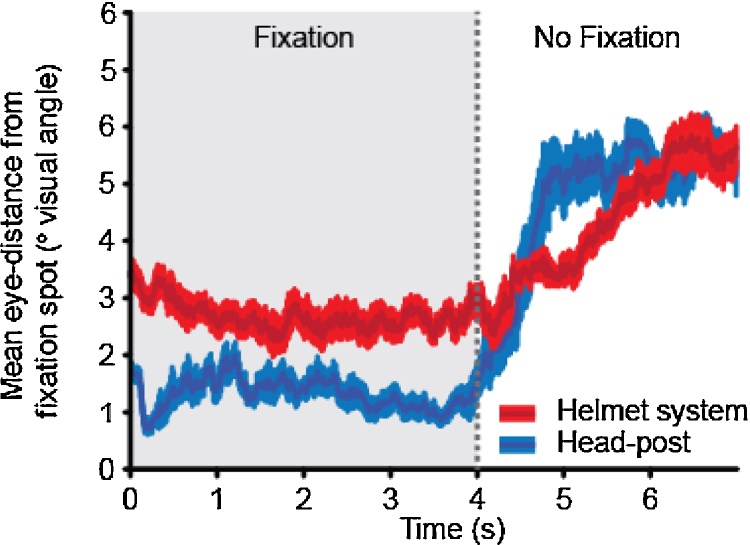
Comparison of eye-tracking data acquired in M3 with implanted head post and the helmet system. The mean distance between the monkeys eye position and the centrally located fixation spot (±SEM) within a 5° fixation window rejection area was calculated during the 4 s fixation period and the subsequent 3 s period during which no stimuli were present and the animal was free to look around.

To assess the stability during a simple eye-fixation task conducted with the helmet system, we compared data gathered from M3 when he was tested using a surgically implanted head post with data gathered using the helmet system. To assess how well M3 fixated, in relation to his prior training and testing with his surgically implanted head post, we initially calculated the variability in looking at the fixation spot as the average distance between his eye position and the fixation spot throughout each trial including 4 s of fixation and the following 3 s of silence where he was not required to fixate. A comparison of eye tracking data acquired with both methods is shown in Fig. 12. While M3 appeared to fixate more tightly using the head-post system (*t*-test comparing the average eye position of the animal during fixation period; *t*_18_ = 7.37, *p* < 0.001), both methods showed significantly less eye movement during the fixation period than during the non-fixation period (*F*_1,36_ = 190.35, *p* < 0.001, helmet system: *t*_18_ = 6.96, *p* < 0.001). It is, however, important to note that while this level of fixation is adequate for our experiments, where we needed general fixation followed by unrestricted viewing towards the location of sound sequences (Wilson et al., 2013), further assessment is required before this method could be applicable to visual research where tighter levels of fixation (< 1° visual angle) are required. It is possible that the additional movement during fixation may be a combination of some movement in the helmet system and an animal that is not fixating as well. Since we see somewhat comparable levels of eye movement for both methods after the fixation period ended, this may suggest that the variation has more to do with the animal’s performance than head movement. In any case, combing eye-tracking with MRI (the latter of which can provide a measure of head movement in the helmet system, as we show below) could help to tease apart the differential contributions to eye fixation performance.

### 3.6 Movement within the helmet measured with MRI

**Fig. 13:**
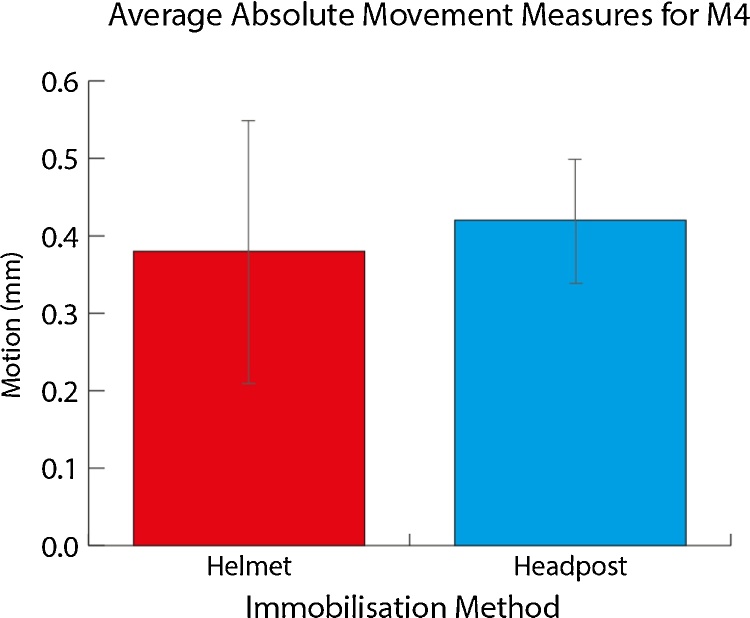
Comparison of movement measures using headpost versus helmet: within animal comparison. Thirty scanning runs of 70–100 imaging volumes each were compared between the two immobilisation methods: with helmet or implanted head post. No significant difference was seen between the two methods for head immobilisation in this animal.

In order to compare levels of movement in the helmet in relation to head posts, we used the MCFLIRT motion detection algorithm in FSL (Jenkinson et al., 2002) after acquisition of functional Echo Planar Imaging (EPI) scans taken with the animals in the scanner awake and being stimulated passively or performing an auditory task. We adapted the helmet system for monkey M4 who had an existing implant and had previously had a large amount of experience with MRI data acquisition. We modified the helmet so that it would immobilise the head without making contact with the implant. Thirty scanning runs of 70–100 volumes (TR 2000 ms, TE 21 ms, flip angle 90°, matrix 92 × 92, field of view 11 cm, number of slices 24, slice thickness 2 mm) were collected over 10 scanning sessions with the animals awake (full head immobilisation durations typically of 1.5–2 h). Motion induced effects on the images were measured using FSL MCFLIRT. The resulting comparison of the movement measures taken using the helmet with the equivalent number of scanning runs using the head post are shown in Fig. 13. An independent samples *t*-test showed that for this animal there was no significant difference between the motion recorded using the two methods (*t*_58_ = 1.11, *p* = 0.27; mean movement in helmet: 0.37 mm, mean movement with head post: 0.42 mm).

**Fig. 14:**
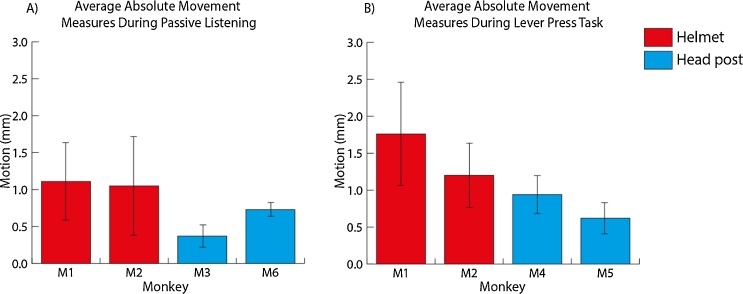
Comparison of movement measures between the NHIS and the headpost on two separate tasks. MRI-based data from initial scanning sessions were taken for each animal to compare across methods (during passive listening: *N* = 11 runs for M1–M3, and 9 for M6; for the active task with lever presses: *N* = 17 runs for all four animals).

Movement measures from four additional animals were also compared as follows: two were immobilised using the helmet system and two others using their implanted head posts. Movement measures in all four animals were taken from initial scanning sessions (i.e., the animals were less familiar with the scanner environment) under similar recording procedures (see Fig. 14A). The four monkeys were scanned during passive listening, M3 and M6 head immobilised with their implanted head posts and M1 and M2 with the NHIS. In these comparisons, there was less head movement in the animals using the implanted head posts (*t*_40_ = 4.3, *p* < 0.001; mean movement in helmet: 1.05 mm; mean movement with head post: 0.53 mm).

We also conducted further comparisons with four animals performing an active auditory task. M1, M2, M4 and M5 had all been trained to perform a lever press task in the scanner, allowing us to compare the head movements in two animals with an implant (M4 and M5) with two animals using the NHIS (M1 and M2) see Fig. 14B. A significant difference was seen between the two methods, with less movement for the implanted head post (*t*_66_ = 5.7, *p* < 0.001; mean movement, helmet: 1.46 mm; head post: 0.78 mm). Although the MRI-based movement with the helmet system can be comparable (in some animals; Fig. 13) or higher than with a surgically implanted head post (between animals; Figs. 14–15), the movement measures we recorded rarely fall outside of 2 mm, a commonly used movement threshold for identification of images that cannot be easily corrected with the latest movement correction algorithms and should be discarded (Wylie et al., 2014). Also from our experience, the EPI quality and distortions are considerable with over 2 mm of motion and even current motion correction algorithms cannot correct for such high levels of head movement. Moreover, as we see with M4 which provided the data for a within monkey comparison of the different types of head immobilisation approaches (Fig. 13), in animals which have further training with MRI scanning, there can be less movement during scanning. Thus, sufficient training time can be a critical variable in minimising head movement within the helmet during MRI.

During fMRI data collection, where testing sessions are longer (∼1 h setup time; 2 or more hours of scanning/testing) potential increases in temperature inside the helmet become a concern due to the less efficient air circulation inside the bore of the magnet. Therefore, for our longer MRI sessions we also placed gel cooling packs behind the animal’s head in a position that would not interfere with the setup. This seemed to be an effective solution for reducing the temperature in the helmet during these sessions. The cooling pack in combination with some of the other approaches described (such as placing additional ventilation holes in the helmet), are promising for progressing to longer testing sessions. However, the use of the system with full head immobilisation testing sessions longer than what we tested (i.e., >2 h) would need to be assessed.

### 3.7 Monetary cost of procedures

We compared the monetary cost of the surgical implant with non-invasive head immobilisation procedures in our facility. We collated representative data on our cost for a surgical implant procedure, maintenance of the skin margin and the implant post-surgery maintenance. The costs for these are compared with those for the procedures required for producing the helmet. An itemised list of the costs is shown in Table 3. The initial setup for the helmet production, including equipment and consumables, is £2111 GBP ($3223 USD). After the initial equipment investment, a large number of helmets can be produced at a cost of approximately £139 ($212) per helmet. In comparison, a single implant procedure costs ∼£1919 ($2930), with additional costs of ∼£145 ($202) for each implant maintenance procedure.

The cost of two replacement helmets per year for a period of four years would be £588 ($898). In contrast, a surgical implant procedure, with 4 implant maintenance procedures per year (average for the animals listed) over the four years would, in our lab, amount to £4239 ($5924). Therefore, under similar conditions to those used in our facility, there is a clear financial benefit to the use of this system.

## 4 Discussion

We developed an individually customised non-invasive head immobilisation system for non-human primates. The approach includes the use of a facemask, which we show to be useful for habituation and initial behavioural training to help reduce animal distress in using the system. The option for automated voluntary facemask engagement can be separately used where full head immobilisation is not required or in combination with whole-head immobilisation to help the animals to habituate to using the full system. Using a relatively inexpensive device for thermal imaging, we also developed an approach for monitoring and preventing pressure point formation, which, since we encountered no occurrences of pressure points, seemed to be successful. We also demonstrate the feasibility and quality of the data for auditory behavioural experiments and for basic eye-fixation training, which for our purposes with auditory tasks does not require sub-degree visual angle fixation. Overall, the system is robust, versatile and can be flexibly incorporated into a number of laboratory setups, being easily modified to suit the individual animals and the requirements of the experimental procedures. We summarise the results obtained with the system in relation to the scientific and animal welfare criteria identified in Table 1.

1) Customisable: The system is individually customised for each animal. It is produced by creating a model of the animal’s head and using this to create a thermoplastic facemask and shell. The aim of individualising the facemask and helmet for each monkey is to improve the fit for each animal (an important feature in recent systems, see Table 1). As we also show, this is not a cumbersome process in terms of cost or the time needed to create the facemask and helmet, even if a replacement of these might be needed. The other aspects of the system (attachments, fittings etc.) can be created once and used with different animals’ facemasks or helmets.
2) Access: The system ensures access for auditory and visual stimulation as well as the delivery of fluid to the animal. Due to the versatility of the system, adjustments can easily be made to the plastic material to allow access to the ears, eyes and mouth.
3) Minimising pressure points: At no point did any animals experience pressure sores as a result of the helmet. The system successfully strikes a balance between coverage of an area large enough to distribute pressure around the head, allowing sufficient spread of pressure across a relatively large area while allowing enough room to modify as needed to open up air holes and spaces to improve the comfort of the animal. Additionally, we were able to monitor the fit of the head immobilisation device with thermal imaging, highlighting areas where the helmet may be too tight. We also show an approach for monitoring hot spots using thermal imaging data to reduce pressure points and sores from forming.
4) Comparisons to implanted head posts: Behavioural performance data, even on difficult auditory tasks, were encouraging in the two monkeys tested (M1–M2) during performance while using the facemask or full helmet. The habituation time for achieving immobilisation for >30 min is comparable in the 5 animals tested in relation to the use of a surgically implanted head post. Head movement with MRI-based measurement within the helmet can be comparable to that of an implant, as it was in M4 (Fig. 13), or up to double that of the stability available with an implant in less experienced animals. Further assessment is required for comparison of the quality of functional MRI (fMRI) data under both conditions as our analysis has so far only used the MRI images to address movement in the helmet system. Finally, additional assessment is needed before the system can be recommended for tasks which require more rigorous control over eye-movements than what was required for our purposes.
5) Minimise distress: There were no obvious behavioural signs of distress exhibited by the animals and we provide evidence that habituation to the device has no lasting effect on performance. Habituation was aided by the transparent plastic, allowing the animal to see through the mask, drawing their attention to rewards rather than the enclosure.
6) Adaptable: The system is highly adaptable and we show how it can be implemented in a more common laboratory testing chair (such as those produced by Crist Instruments or Rogue Research). We also show it in place with modifications to the fixation points in a more specialised MRI setup in use at Newcastle (a vertical bore primate-dedicated 4.7 T MRI scanner) and that it can be modified to immobilise an implanted animal without contacting the implant. This modification demonstrates the potential for easy modification for electrode placement for electroencepholagraphic (EEG) recording, or to incorporate a chamber for direct neuronal recording. The quality of the data achievable with these methods would need to be separately evaluated as it was beyond the scope of the current report. In the more immediate future we plan to evaluate the system for use with functional MRI, including systematic assessment of the quality of fMRI data in relation to those obtained with surgically implanted head posts.
7) Voluntary engagement: A key feature of the system is its ability to be used as a facemask attached to any training setup. The facemask can incorporate a sensor to automatically identify if the animal has engaged the facemask, at which point a reward is provided to encourage longer periods of self-immobilisation. This can expedite training and the transparent nature of the mask allows the animal to see rewards through the plastic, encouraging continuing engagement to obtain the reward. The use of a sensor to detect the face during habituation was adequate for our purposes, but the option also exists for the use of eye trackers to automate identification of engagement with the facemask in combination with a visual task (Kiorpes et al., 2012, Fairhall et al., 2006). We have confirmed that the animals will readily engage with the mask for reward and showed progression from habituation to full immobilisation with animals working on different types of tasks. Thus, the two parts of our system (the facemask and the helmet) can be used flexibly as needed and can be used in conjunction with training and behavioural data collection on tasks, even while the animals habituate to the helmet system. The system may also be useful for increasing the training potential in animals, such as those more prone to moving their head around and not attending to the sounds or screen in front of them. Using the facemask alone in this case can also mean that the animal self immobilizes by placing its face in the facemask while working on the task. This would potentially increase the quality of the auditory or visual behavioural data than if the animal is free to move its head around (see Fig. 7).
8) Larger animals: We have shown that the system works well with larger animals (8–16 kg) and is therefore likely to be a viable method for use with most rhesus macaques, and possibly other species of primates, although this would need to be separately tested.

In addition, over a one year period we monitored the number of sedations required to obtain a model of the head for the non-invasive head immobilisation system and compared that to animals that required sedation to maintain or monitor surgical implant stability. Consistently, animals with the non-invasive helmet required 2–3 sedations. In contrast, the number of sedations for implanted animals ranged from 0 to 8 during a one year period. In addition, since the head impressions can be obtained relatively quickly (15–25 min), they could be combined and obtained during other planned sedation or veterinary procedures.

Finally, we have shown that implementation of this NHIS is cost effective for our lab. The combination of implant and implant maintenance costs are much higher than the cost of producing multiple helmets, once the required equipment and materials for the helmet system are in place.

## 5 Conclusions

We designed and systematically tested an individually customisable non-invasive head immobilisation system which is robust and flexible to implement, and, for the first time, combines a facemask with a whole head immobilisation approach providing the option for voluntary engagement. Additionally, an approach was developed for monitoring hot spots with thermal imaging, to prevent pressure points or sores from forming. We also evaluated the system with behavioural tasks and MRI-based head movement measures. Moreover, we show that the system is not time consuming to create, generally does not take much longer to train the animals to use and is far cheaper to implement than traditional surgical implant approaches. The system offers the opportunity to conduct non-invasive scientific experiments with head immobilisation, while reducing the reliance on surgically implanted head posts and improving animal welfare. This work and that of other recent efforts provide information that may be useful for laboratories to consider as they weigh the costs and benefits of using non-invasive head immobilisation for certain procedures.

## Funding

This project was initially funded by the NC3Rs (CIP & HS, Pilot Grant). It was further supported by the Wellcome Trust (CIP, Project Grant WT092606/Z/10/Z; Investigator Award WT102961MA), and BBSRC (CIP and Quoc Vuong, BB/J009849/1). The funding bodies had no input in the design of the study, data collection, analysis or interpretation, and were not involved in the writing of the report nor the decision to publish.

## Author contributions

H.S. and C.I.P. designed the system; H.S. and A.M. performed data collection; R.M. assisted with thermal measurement and mould procedures; H.S and BW analysed data; F.B and D.H assisted with MRI imaging; all authors provided intellectual contributions or materials. H.S. and C.I.P. wrote the paper.

## Figures and Tables

**Table 1:** Recent developments in Non-invasive Head Immobilisation Systems (NHIS) for macaques and key features.

	Customisable	Access	Minimise pressure points	Tested with:	Minimal distress	Lab adaptable	Voluntary engagement	Animal sizes
Howell et al. (2001)*	✓	×	?	PET	✓	?	×	6–11 kg
Amemori et al. (2015)*	✓	×	?	Ephys, TMS	?	×	×	6–7 kg
Itoh et al. (2015)*	×	✓	?	EEG	?	?	×	5–8 kg
Drucker et al. (2015) +	✓	?	?	Eye tracking, Ephys	?	?	×	5–13 kg
Machado and Nelson (2011) +	✓	✓	?	Eye tracking	✓	?	×	10–14 kg
Srihasam et al. (2010) ★	✓	✓	?	fMRI	?	×	×	5–10 kg
Hadj-Bouziane et al. (2014) ★	✓	✓	?	fMRI	?	×	×	5–6 kg
Fairhall et al. (2006) ♦	✓	✓	✓	Eye measurement	✓	×	✓	9–11 kg
Kiorpes et al. (2012) ♦	✓	✓	✓	Eye tracking	✓	×	✓	?

Columns identify eight scientific and animal welfare needs in relation to recent NHIS. These are also used to develop and evaluate a new system. Customisable: cannot be ‘one-size-fits all’. Access for the animals to hear, see and obtain rewards. Minimise pressure points: to avoid pain, sores and infection. Tested with: seems to offer comparable head immobilisation to surgically implanted approaches for certain applications, see cited papers for details. Minimal distress: should minimise distress during immobilisation. Lab adaptable: to a variety of settings/setups. Voluntary engagement: option for voluntary engagement with the system, to help with habituation and minimise distress. Animal size: should work with a broad range of small (5–6 kg) to large and strong animals (>10 kg). Symbols identify similar approaches: * involves the use of foam to hold the head in place either encapsulating the animal’s head (Howell et al., 2001) or providing support to a specific area of the head to reduce movement (Amemori et al., 2015, Itoh et al., 2015); + use a heated mesh of thermoplastic material that is drawn over the animal’s head to form a helmet as the mesh cools (Machado and Nelson, 2011, Drucker et al., 2015). ★ and ♦ use an impression taken of the animal’s head, e.g., with MRI, to create a model which is used to make the system. In ★ the head model is used to create a cap that attaches to vacuum suction system and has a chin strap to further reduce head movement (Srihasam et al., 2010, Hadj-Bouziane et al., 2014). ♦ transparent facemask using thermoplastic material vacuum formed around a model of the animal’s head (Fairhall et al., 2006, Kiorpes et al., 2012); these options have not yet been incorporated into full-head immobilisation.

**Table 2:** Summary of the procedures on which each animal was tested.

	M1	M2	M3	M4*	M5*	M6*	M7*
MRI for 3D head model (Fig. 3)	✓	✓	✓				
Auditory task performance (Fig. 7)	✓	✓					
Habituation time to immobilisation (Fig. 8)	✓	✓	✓	✓	✓	✓	✓
Thermal imaging (Fig. 9)	✓	✓	✓				
Number of sedations (Fig. 10)	✓	✓	✓	✓	✓	✓	✓
Simple eye-tracking (Fig. 12)			✓				
MRI head movement (Fig. 13, Fig. 14)	✓	✓	✓	✓	✓	✓	

Asterisks indicate animals with surgical implants that were used to compare results to those obtained from animals using the NHIS.

**Table 3:** Comparison of monetary costs for surgical implant procedure and helmet production.

Helmet	
Plastic	£5/sheet
One off (initial setup)	
Vacuum former	£1200
Thermal camera	£796
Frame for square chair	£15 materials
£100 labour
Consumables	
Plaster	£20 for 25 kg
Alginate	£30 for 2 kg
Bandages (per procedure)	£1.00
Ketamine	£12/10 ml
Oxygen	£25
Use of prep room	£100
Other	
Vets/Theatre cost	£100.00
Anaesthesia	£65
Scanner	£295/h
3D models	$910 (£544) each
Total for equipment:	£2111
Total for MRI procedure:	£1004
Total for impression mould procedure:	
Basic:	£139
With Alginate:	£147
Surgical implant	
PEEK post	£40
Workshop costs	£100
Theatre cost (1 day)	£822
Vet costs	£232
Dental acrylic	£65
MRI compatible screws	£400
Consumables and post-op care (analgesia etc.)	£259
Total for surgical implant procedure:	£1918
Implant maintenance	
X-rays	Husb/Vet
Chemical cauterization	Husb/Vet
Dermasol cream	£8
Wonder dust	£17 (per 113 g bottle)
Ketamine	£12/10 ml
Other consumables	£25
Oxygen	£25
Use of prep room	£100
Total for implant maintenance	£145

Husbandry costs are not included. Anaesthesia refers to procedures following short term ketamine sedation.
