# Supplementary material for: Individually customisable non-invasive head immobilisation system for non-human primates with an option for voluntary engagement
Source: J Neurosci Methods. 2016 Aug 30;269:46–60. doi: 10.1016/j.jneumeth.2016.05.009 (PMC4935671; doi:10.1016/j.jneumeth.2016.05.009)
Supplement: Supplementary file 1 [file mmc1.pdf]

**Supplementary Materials for Slater et. al., '***Individually Customisable Non-Invasive Head Immobilisation System for Non-Human Primates with an Option for Voluntary Engagement***'**

## I. **Rendering_M2.mpg.** Associated with Fig. 3

After image acquisition the scans are processed and converted to a 3D surface using Amide 3D software. This video shows the full head 3D image which is then sent to a 3D printer for creating the physical model of the head that is used to create the facemask and helmet.

## II. **M2_Voluntary_engagement.3GP.** Associated with Fig. 6

Initial stages of habituation involve holding the translucent mask or anchoring it to the chair while encouraging the animal to receive rewards (in this case juice) through the mouth piece. In this way the animal is focussed on the positive reinforcement while simultaneously becoming accustomed to the presence of the mask and placing its head into it to get the reward.

## III. **M3_Voluntary_Engagement.mp4.** Associated with fig. 6

Following the initial presentation of the mask to the animal as in the previous video, the mask is fixed to the training chair via the custom made frame and the sensor (white on top left of the mask) is placed to automatically allow the experimental system to provide fluid rewards whenever the animal places their face within the mask. Note that for some of the self-initiated trials the animal can self-restrain for many seconds and the experimental software can regulate the reward to encourage longer periods of self-restraint.
