# Supplementary figures and images for: Individually customisable non-invasive head immobilisation system for non-human primates with an option for voluntary engagement
Source: J Neurosci Methods. 2016 Aug 30;269:46–60. doi: 10.1016/j.jneumeth.2016.05.009 (PMC4935671; doi:10.1016/j.jneumeth.2016.05.009)

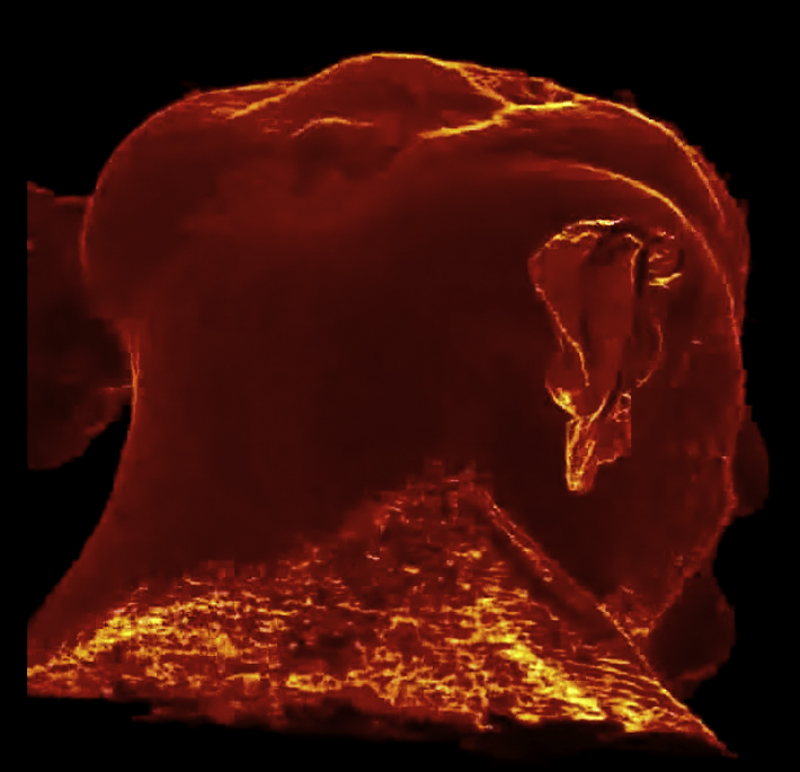

Supplement: Supplementary file 2 [file mmc2.jpg]

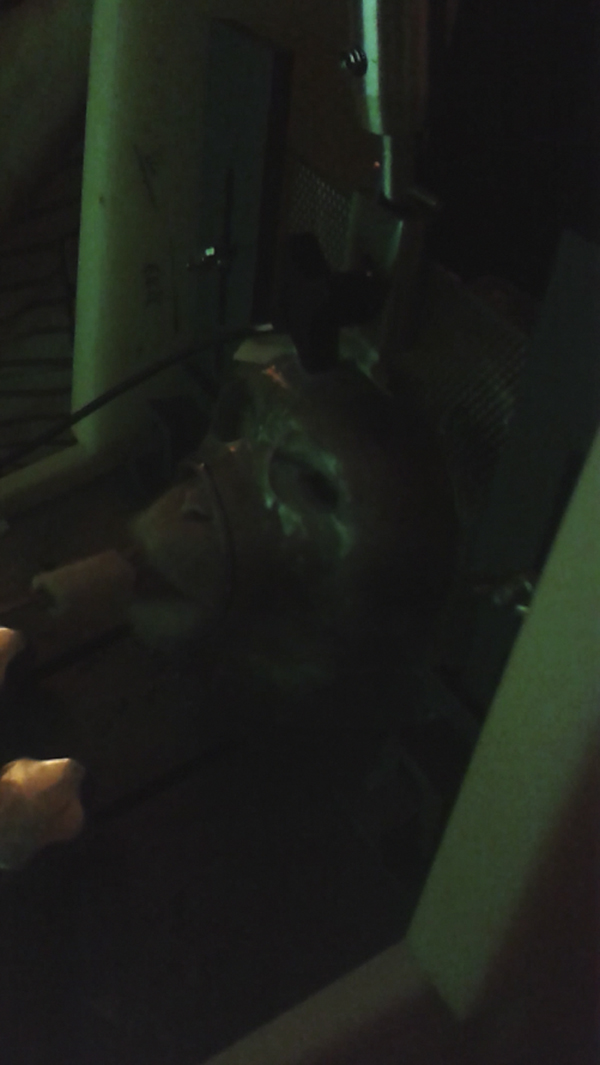

Supplement: Supplementary file 3 [file mmc3.jpg]

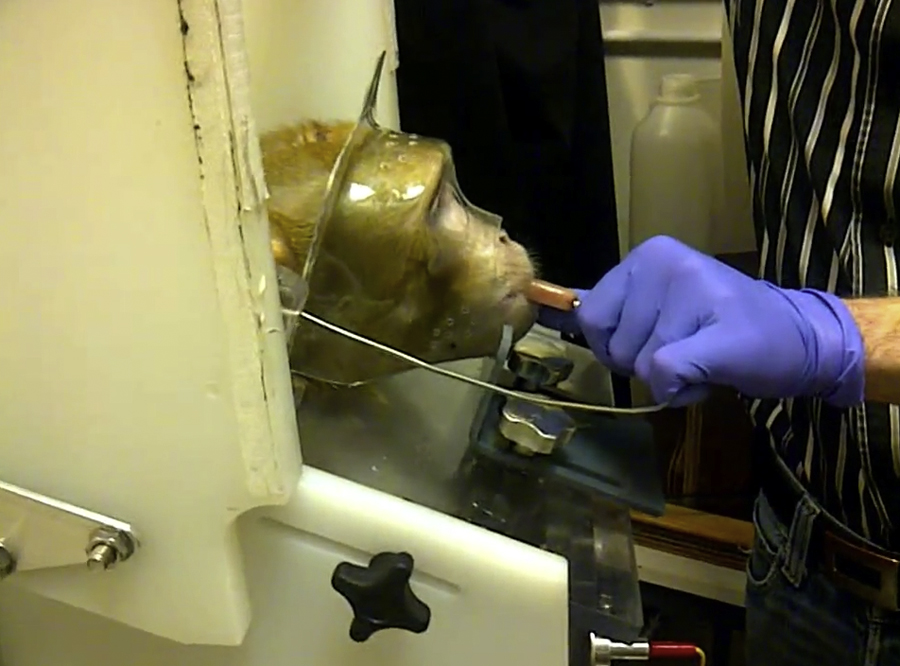

Supplement: Supplementary file 4 [file mmc4.jpg]
